# Common Antacid Medication–Ranitidine Causing a Rare Serious Adverse Effect

**DOI:** 10.7759/cureus.3578

**Published:** 2018-11-12

**Authors:** Simcha Weissman, Michael Siu, Samson Ferm, Ammar Hassan

**Affiliations:** 1 Internal Medicine, Touro College of Osteopathic Medicine, New York, USA; 2 Internal Medicine, New York-Presbyterian / Queens, New York, USA; 3 Gastroenterology, Hackensack University Palisades Medical Center, Lodi, USA

**Keywords:** stomach acidity, antacid, anaphylaxis

## Abstract

Ranitidine is a widely used over-the-counter antacid medication, and is generally very well tolerated. To our knowledge, anaphylaxis secondary to ranitidine is exceedingly rare, and has only been reported in a few case reports. We present a patient who developed an anaphylactic reaction after one tablespoon of ranitidine. The goal of this manuscript is to add to the paucity of literature of this rare but life-threatening adverse effect of a commonly used medication.

## Introduction

Ranitidine is a commonly used, over-the-counter medication, prescribed by clinicians all across the world. Serving as an anti-histamine, ranitidine competitively inhibits the H2 receptor on gastric parietal cells, thus inhibiting the action of histamine, a potent stimulator of acid secretion [1]. The primary use of this medication is to relieve symptoms associated with erosive esophagitis (EG), gastro-esophageal reflux disease (GERD), and Zollinger Ellison (ZE) syndrome. While some side effects have been reported, they are relatively minor, harmless, and certainly non-life-threatening [1]. We present a 34-year-old male patient, with no previous history of allergies to any medication who presented with chest tightness, difficulty breathing, and a diffuse rash within minutes after taking ranitidine for epigastric discomfort. There are only a handful of cases reported in the literature that describe an anaphylactic reaction to this medication. Of those cases reported, it occurred in either an obstetric patient, intra-operatively, or someone with prior allergies to multiple medications. Our case is unique in that our patient had no past history of any drug allergies and his method of administration was oral. To our knowledge, this is the first reported case in the United States, in which a patient without any previous history of allergies presents with severe anaphylaxis after taking an orally administered dose of ranitidine.

## Case presentation

A 34-year-old male with a past medical history of hypertension, presented to the hospital with a chief complaint of diffuse rash, chest tightness, and difficulty breathing. Earlier that morning, he was experiencing stomach pain, nausea, and diarrhea prompting him to take one tablespoon of his wife’s liquid ranitidine in an attempt to alleviate his symptoms. Approximately 15 minutes after ingestion of the liquid ranitidine, he noticed a diffuse body rash, itching, as well as what felt like throat tightening. He went to a local urgent care and was given diphenhydramine 50 mg, prednisone 60 mg, albuterol inhalation, and epinephrine 0.3 mg intramuscularly. Although his breathing improved he was sent to the emergency department for further evaluation. Soon after arriving in the emergency department, he started feeling a recurrence of his chest and throat tightness. Vital signs at the time were within normal limits and he was saturating at 95% on room air. His physical examination was unremarkable. Routine blood work was significant for white blood cell count of 12.07 K/uL. A chest X-ray ordered showed clear lungs, and his electrocardiogram was normal. He recalls a similar incident a few years ago after taking ranitidine, where he developed a diffuse pruritic rash but denied any difficulty breathing or any throat tightness. He was admitted for monitoring of his anaphylaxis to ranitidine and was given 1-liter normal saline bolus, methylprednisolone 40 mg, and diphenhydramine 50 mg intravenously. The patient's breathing remained stable and his rash improved. His leukocytosis was attributed to the steroids and thus not a concern. He was discharged home later that day to follow up with his primary physician. Additionally, and perhaps most importantly, he was strongly cautioned and warned to avoid taking ranitidine in the future.

## Discussion

Ranitidine—a potent inhibitor of the H-2 receptors within gastric parietal cells—is one of the medications used to help reduce acidity of the gastric lumen [1]. Moreover, ranitidine is typically used as an agent to treat allergic reactions, the very condition that it precipitated in this case. Tiredness, dizziness, headaches, and gastrointestinal manifestations have all been previously described in literature as common adverse effects. Additionally, cardiovascular, hepatic and neuropsychiatric complications can occur [1]. Rarely, however, does severe hypersensitivity to this medication occur. It is important to define anaphylaxis, in order to differentiate it from an allergic response. True anaphylaxis is a clinical symptom, often life-threatening, that causes respiratory and/or cardiovascular compromise. Initial symptoms may include angioedema, laryngeal edema, as well as bronchospasm with possible skin manifestations. While anaphylaxis cannot be definitively diagnosed by a single test, clinical symptoms are often diagnostic. A positive skin prick test, oral challenge, or histamine release test, can also be suggestive, but are often unnecessary to establish a hypersensitivity to a reactant. Currently, it is not well understood how ranitidine causes anaphylaxis. One proposed theory, for its pathogenic mechanism, is perhaps that serum specific Immunoglobulin E is present in both ranitidine and human serum albumin, causing true anaphylaxis [2]. Thus, upon encountering a drug with capability to cause an anaphylactic reaction, pro-inflammatory mediators are released from both mast cells and basophils, inducing the severe reaction [3].

The main armamentarium to decrease stomach acidity are the H2 blockers and proton pump inhibitors. H2 blockers are a less expensive option for the management of GERD, ulcers, and other common culprits of dyspepsia. However, proton pump inhibitors, such as omeprazole, are considered superior in terms of efficacy. Nonetheless, maintenance therapy with drugs that act along the same pharmacologic pathway as ranitidine remains indicated for patients at risk for disease recurrence [4]. Considering both drug classes, adverse effects of H2 receptor antagonists and proton pump inhibitors account for only 0.2%–0.7% of all reported cases of anaphylaxis [5].

## Conclusions

In conclusion, anaphylaxis should be considered if swelling, hives, difficulty breathing, and/or chest tightness, develop within temporal proximity to intravenous or even oral administration of ranitidine. Additionally, clinicians should be aware of this reaction as to not overlook H2 blockers as the culprit when treating anaphylaxis. Furthermore, anaphylaxis should be considered even in a patient without any prior allergic history. Hence, what is typically considered a benign medication could lead to fatal outcomes if awareness of this rare, but serious reaction is not raised.
